# Postoperative Bloodstream Infection Is Associated with Early Vascular Complications in Pediatric Liver Transplant Recipients with Biliary Atresia

**DOI:** 10.3390/jcm12216760

**Published:** 2023-10-25

**Authors:** Ho Jong Jeon, Ji-Man Kang, Hong Koh, Myoung Soo Kim, Kyong Ihn

**Affiliations:** 1 Division of Pediatric Surgery, Department of Surgery, National Health Insurance Service Ilsan Hospital, Goyang 10444, Republic of Korea; gdijhj@nhimc.or.kr; 2 Department of Pediatrics, Severance Children’s Hospital, Yonsei University College of Medicine, Seoul 03722, Republic of Korea; 3 Institute for Immunology and Immunological Diseases, Yonsei University College of Medicine, Seoul 03722, Republic of Korea; 4 Division of Gastroenterology, Hepatology, and Nutrition, Department of Pediatrics, Severance Hospital, Yonsei University College of Medicine, Seoul 03722, Republic of Korea; 5 Department of Surgery, Yonsei University College of Medicine, Seoul 03722, Republic of Korea; 6 The Research Institute for Transplantation, Yonsei University College of Medicine, Seoul 03722, Republic of Korea; 7 Division of Pediatric Surgery, Department of Surgery, Severance Children’s Hospital, Yonsei University College of Medicine, Seoul 03722, Republic of Korea

**Keywords:** bacteremia, vascular complication, pediatric liver transplant, biliary atresia, bloodstream infection

## Abstract

Bloodstream infection (BSI) after pediatric liver transplantation (PLT) is a common and severe complication that affects patient survival. Children with biliary atresia (BA) are at an increased risk for clinically significant infections. This study evaluated the impact of post-PLT BSI on clinical outcomes in children with BA. A total of 67 patients with BA aged <18 years who underwent PLT between April 2006 and September 2020 were analyzed and divided into two groups according to the occurrence of post-PLT BSI within 1 month (BSI vs. no BSI = 13 [19.4%] vs. 54 [80.6%]). The BSI group was significantly younger at the time of PLT and had a higher frequency of BSI at the time of PLT than the no BSI group. Early vascular complications within 3 months and reoperations were significantly more frequent in the BSI group. Univariate and multivariate analyses revealed that bacteremia within 1 month of PLT and graft-to-recipient weight ratio >4% were significantly associated with vascular complications. In conclusion, BSI after PLT is associated with increased vascular complications and reoperations. Proper control of bacterial infections and early liver transplantation before uncontrolled BSI may reduce vascular complications and unexpected reoperations in children with BA.

## 1. Introduction

Biliary atresia (BA) is a rare condition characterized by the progressive development of liver cirrhosis in early infancy owing to irreversible fibro-obliterative changes in the intrahepatic and extrahepatic biliary tracts [1]. The primary treatment for BA is Kasai portoenterostomy (KPE), which aims to restore bile flow into the intestine, thereby preventing or slowing down the progression of liver injury and cirrhosis [2]. Despite the initial success of KPE in restoring bile flow in most infants with BA, it is the leading diagnosis in approximately 30–50% of pediatric liver transplantation (PLT) cases [3].

One significant indication for liver transplantation in patients with BA is intractable recurrent cholangitis, a condition characterized by recurrent episodes of cholangitis despite aggressive antibiotic therapy, life-threatening sepsis, the presence of multidrug-resistant organisms, or severely impaired quality of life due to recurrent or prolonged hospitalization [4]. Even when liver transplantation is performed when bloodstream infection (BSI) has already progressed, many patients succumb to persistent systemic infections, resulting in a low survival rate after transplantation [5,6,7,8].

Vascular complications after PLT are among the most critical challenges that threaten graft and patient survival, with a higher incidence in pediatric patients than in adults [9,10]. Early complications such as hepatic artery or portal vein thromboses are often attributed primarily to surgical factors such as anastomotic stenosis, vessel diameter, graft size mismatch, and angulation [3,11]. However, recent research has focused on nonsurgical factors, such as coagulation abnormalities, infections, and immunologic imbalances, which contribute to these complications [12]. Bacterial toxins have been confirmed to directly damage endothelial cells, playing a crucial role in the induction of intravascular coagulation and other vascular dysfunctions [13,14,15,16,17]; however, comprehensive studies investigating the potential association between BSI and the emergence of early vascular complications after PLT are lacking. Hence, the present study aimed to evaluate the impact of post-PLT BSI on the clinical outcomes in children with BA. We hypothesized that BSI might be a risk factor for early vascular complications, thus impairing the outcomes in children with BA undergoing PLT.

## 2. Materials and Methods

### 2.1. Study Design and Patients

The institutional review board of Severance Hospital, Yonsei University Health System (IRB number: 4-2020-1015) approved this study. We enrolled all PLT recipients aged <18 years who had a primary diagnosis of BA between April 2006 and September 2020 at Severance Hospital, Yonsei University College of Medicine, amounting to 70 patients. Patients who underwent retransplantation or simultaneous transplantation of additional organs were excluded. Additionally, two patients who developed BSI after experiencing early vascular complications were excluded to ensure a causal relationship between BSI and vascular complications. Finally, 67 patients were included in the study and were followed up for at least 24 months after transplantation.

An electronic medical database was used to record various data, including age, sex, pediatric end-stage liver disease (PELD) or model for end-stage liver disease (MELD) scores, history of BSI before PLT, ongoing infections at the time of PLT, the occurrence of BSI after PLT, operative complications, acute rejection episodes, infection sites, history of reoperation, and status of graft or patient survival. The incidence of early vascular complications was assessed within 3 months of PLT. Recipients who experienced post-PLT BSI (BT) within 1 month were compared to those without post-PLT BSI (NBT). The primary outcomes of interest were risk factors associated with early vascular complications after PLT, whereas the secondary outcomes included overall patient survival in the BT and NBT groups.

### 2.2. Surgical Technique

Liver grafts were procured from living (68.7%) or deceased (31.3%) donors. The graft types consisted of the left lateral segment (LLS, 77.6%), left lobe (11.9%), right lobe (4.4%), reduced LLS (1.5%), and the whole liver (1.5%). Total hepatectomy was performed using conventional techniques in all the cases. The recipient’s vena cava was preserved, and a piggyback technique was used without venovenous bypass. The left hepatic vein of the graft was anastomosed to the confluence of the recipient’s left, middle, and right hepatic veins or the left–middle hepatic vein confluence. In cases where a right-lobe graft was used, the right hepatic vein of the graft was anastomosed to the recipient’s right hepatic vein using continuous 5-0 non-absorbable monofilament sutures. Artificial or cadaveric vessel conduits have been used as interposition grafts to drain the major middle hepatic vein tributaries. When the donor and recipient portal veins were size-matched, portal vein reconstruction was performed with end-to-end anastomosis using continuous 6-0 non-absorbable monofilament sutures posteriorly and interrupted sutures anteriorly. Reconstruction was performed via vein graft interposition or renoportal anastomosis in selected cases in which the donor and recipient portal veins were not size-matched or healthy. Portosystemic shunt ligation was performed in the recipients with inadequate portal flow. After graft reperfusion, hepatic arterial anastomoses were completed in an end-to-end fashion using 8-0 or 9-0 non-absorbable monofilament sutures aided by a surgical microscope (under ×10 magnification) or surgical loupes (under ×5 magnification). Because of the previous KPE surgery, bile duct reconstruction was performed as a hepaticojejunostomy with a Roux-en-Y limb using absorbable monofilament sutures. In cases with multiple bile ducts, the distance between the donor bile ducts was measured to determine whether they were anastomosed separately or conjointly.

The vascular flow of the liver graft was routinely assessed using Doppler ultrasonography after each vascular anastomosis step and before and after abdominal wall closure. Doppler ultrasonography was used to evaluate the vascular patency, flow velocities, and bile duct diameter, with assessments conducted on postoperative days 1, 2, 4, and 7 by a specialist pediatric radiologist. In cases of uncertainty, computed tomography was performed.

### 2.3. Postoperative Immunosuppressive Regimen

The immunosuppressive regimen after PLT consisted of dual therapy with tacrolimus (FK-506) and corticosteroids. Tacrolimus dosages were maintained and adjusted according to blood trough levels, with target trough levels as follows: 10–12 μg/L during the first month, 8–10 μg/L until the third month, and 6–8 μg/L until one year after PLT. Tacrolimus was then gradually tapered to maintain 3–5 μg/L levels during outpatient clinic follow-ups. Basiliximab was administered at the time of PLT and on the fourth postoperative day at a dose of 10 mg/day for recipients weighing less than 35 kg or 20 mg/day for those weighing more than 35 kg. Subsequently, immunosuppression was maintained using tacrolimus and corticosteroids, with the corticosteroids gradually tapered and discontinued within 6–12 months. Mycophenolate mofetil was administered to selected patients who experienced acute rejection at a dose of 20 mg/kg/day for at least 6 months.

### 2.4. Definition and Workup of Bacterial Infection

Bacterial infections were defined according to the criteria set by the Centers for Disease Control and Prevention. Early BSI was defined as a BSI occurring within the first 30 postoperative days. To adhere to the protocol, at least two sets of blood cultures were routinely obtained from the central venous lines until the seventh postoperative day. After this period, blood cultures were drawn in response to the clinical symptoms of infection. Specific pathogens, such as coagulase-negative S*taphylococci*, *Corynebacterium* spp., *Bacillus* spp., *Cutibacterium* spp., non-hemolytic *Streptococci* of the viridans group, *Aerococcus* spp., and *Micrococcus* spp. were regarded as causative agents of BSI only if they were isolated from two or more separate blood cultures in conjunction with clinical signs of infection, such as fever, elevated inflammatory markers, or unstable vital signs. Patients in the BT group were limited to those with clinically suspected infections, as confirmed by blood culture results. Blood samples were processed using BacT/ALERT aerobic and anaerobic medium culture bottles, which were incubated for 5 days in the BacT/ALERT 3D blood culture system. Positive signals from blood culture bottles were followed by Gram staining and culture using blood agar and chocolate agar plates at 35 °C in a 5% CO_2_ incubator. The identification and susceptibility tests for culture-positive cases were conducted using the VITEK 2 system (bioMérieux, Marcy l’ Etoile, France).

### 2.5. Perioperative Antibacterial Protocol

The prophylactic antimicrobial regimen consisted of piperacillin/tazobactam monotherapy administered intravenously every 8 h, with the first dose administered just before the operation and continued for 7 days postoperatively (or longer if the patient’s condition remained unstable). For patients weighing less than 40 kg, 90 mg/kg piperacillin/tazobactam was administered every 6–8 h. In cases of recurrent or intractable cholangitis, the same preoperative effective antibiotics were maintained until the resolution of infectious signs, as per the clinical decision.

### 2.6. Perioperative Management of Coagulopathy

All recipients underwent daily coagulation and thrombophilic evaluation for a week to ensure the levels were close to or within the normal range for protein C, protein S, antithrombin III (AT-III), and fibrinogen. AT-III concentrate was administered when AT-III levels fell below 70%, at doses of 1000 units/day for patients weighing less than 30 kg, 2000 units/day for those weighing 30–60 kg, and 3000 units/day for those weighing over 60 kg. Prostaglandin E1 was used to inhibit platelet aggregation and to vasodilate in cases of reperfusion injury (0.5 mg/kg/day intravenously), with treatment continued for up to 6 days postoperatively before transitioning to acetylsalicylic acid when oral supplementation was feasible. Acetylsalicylic acid (3 mg/kg/day orally) was maintained as long as the international normalized ratio (INR) remained below 1.5 and the platelet count was above 80,000/dL. This regimen was continued for 12 months after PLT.

### 2.7. Statistical Analysis

Baseline characteristics and demographic data are summarized using the number of available data points, patient numbers, percentages, or median values with interquartile ranges. Fisher’s exact test or Pearson’s chi-squared test was used for between-group comparisons of categorical data. Continuous variables with a normal distribution were compared using the Student *t*-test for two independent samples. Wilcoxon rank-sum tests with continuity corrections were used for two-group comparisons when data did not follow a normal distribution. The Kaplan–Meier method was used to estimate graft and patient survival times, and the log-rank test was applied to compare overall patient survival between the groups. Statistical significance was defined as *p*-value < 0.05, with all *p*-values reported for each calculation. Statistical analyses were performed using SAS version 9.4 (SAS Institute, Cary, NC, USA) and SPSS version 26 (IBM Co., Armonk, NY, USA).

## 3. Results

### 3.1. Patient Characteristics

During the study period, 67 PLTs were performed at Severance Hospital. Among these patients, 13 experienced episodes of post-PLT BSI within 1 month, with four patients encountering two such episodes. The baseline preoperative clinical characteristics of the patients with and without post-PLT BSI within 1 month (BT and NBT) are summarized in Table 1. The study population comprised 27 males (40.3%) and 40 females (59.7%), and the median age at the time of PLT was 1.08 (interquartile range [IQR]: 0.67–5.33) years. The median follow-up duration was 45 (IQR: 21–76) months. Compared to the NBT group, the BT patients exhibited a higher prevalence of bacteremia at the time of PLT (15.4% vs. 0%, *p* = 0.035). BT patients had higher white blood cell counts (6330 vs. 10,530, *p* = 0.006) and marker values indicating native liver function, including serum total bilirubin levels (10.9 vs. 6.1, *p* = 0.046), INR values (1.50 vs. 1.26, *p* = 0.039), and MELD/PELD scores (15.4 vs. 9.0, *p* = 0.037) than NBT. However, there were no significant differences in age, sex, body weight at the time of PLT, aspartate aminotransferase and alanine aminotransferase levels, or admission status at the time of PLT between the two groups.

The donor and surgical characteristics of the two groups were compared, as shown in Table 2. LLS was the most frequently used graft type in both groups (77.6%). Donor age was lower in the BT group than in the NBT group (26 vs. 32 years, *p* = 0.039), and the graft-to-recipient weight ratio (GRWR) was significantly higher in the BT patients (3.04% vs. 2.22%, *p* = 0.047). However, the two patient groups showed no significant differences in donor graft weight, proportion of grafts from deceased donors, or complex vascular reconstruction.

### 3.2. Complications and Operative Outcomes

**Figure 1:**
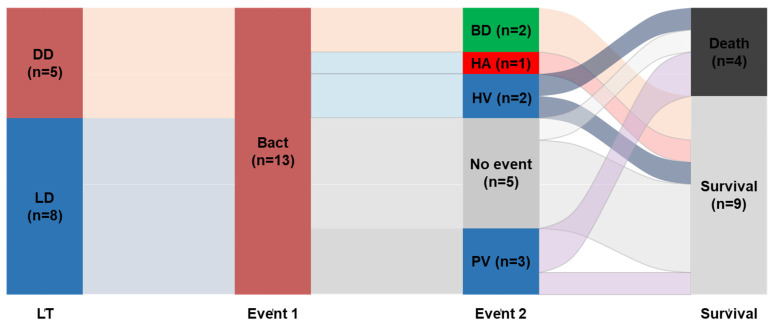
Sankey diagram showing the vascular complications and survival outcomes after PLT in the BT group. Bact, bacteremia; BD, bile duct complication; DD, deceased donor liver transplantation; HA, hepatic arterial complication; HV, hepatic venous complication; LD, living donor liver transplantation; PV, portal venous complication.

Table 3 presents the frequency of early post-transplantation complications (within 3 months) and clinical outcomes. The incidence of vascular complications, regardless of type, was higher in the BT group than in the NBT group (46.2% vs. 18.5%). Among the 13 BT patients, three experienced early portal vein complications after PLT (Figure 1), and two of these children succumbed to infection (one patient exhibited concurrent portal vein stenosis and thrombosis). One patient in the BT group developed hepatic artery thrombosis that was successfully treated with intravenous anticoagulation therapy, resulting in a patent artery. Furthermore, the BT group exhibited a higher incidence of re-laparotomy than the NBT group (30.8% vs. 3.7%, *p* = 0.011). The duration of endotracheal intubation, intensive care unit stay, and hospital stay after PLT were similar between the BT and NBT groups. Similarly, there was no significant difference in survival between the BT and NBT groups concerning the 3-year graft survival (76.9% vs. 80.5%) and 3-year overall survival (84.6% vs. 80.5%).

### 3.3. Bacterial Infection Episodes

Table 4 presents the microorganisms identified in bacterial infections within the BT group. Each pathogen was identified at its source and confirmed as the causative agent responsible for BSI. The analysis revealed the presence of three Gram-positive and seven Gram-negative strains. Eight episodes involved pathogens identified from intra-abdominal drainage tubes and were associated with bacteremia, while in five episodes, pathogens were isolated from vein catheters. Pathways were identified from intra-abdominal drainage tubes with suspected biliary leakage in two instances. Concurrently, one case involved identifying the pathogen in the blood following the sudden onset of gastrointestinal symptoms. The pathogen was linked to a respiratory tract infection in the remaining case.

### 3.4. Risk Factors for Vascular Complications after PLT

Regression analysis was performed with short-term vascular complications as the outcome variable. The results of the univariate and multivariate analyses are presented in Table 5. After adjusting for potential variables that could influence the outcome in a multivariate model, the occurrence of bacteremia within 1 month after PLT (OR 5.691, *p* = 0.041) and GRWR > 4% (OR 27.214, *p* = 0.013) were significantly associated with vascular complications.

### 3.5. Vascular Characteristics of Doppler Ultrasonography before and after BSI in BT Patients

Table 6 shows the vascular characteristics of BT patients both before and after BSI, along with the patients’ BSI pathogens, antibiotic resistance, impact of antibiotic treatment, and presence or absence of vascular complications. BSI occurred a median of 5 (IQR: 3–6) days after PLT. Doppler ultrasonography was performed a median of −2 (IQR: −2 to −1) days before BSI and a median of 2 (IQR: 1–3.5) days immediately after BSI. Among the patients who developed vascular complications, Patient 2 experienced BSI 5 days after PLT. There were no abnormal perfusion findings on Doppler ultrasonography before and immediately after BSI onset. However, PV stenosis and thrombosis subsequently occurred. Patient 3 developed BSI 2 days after PLT. Doppler ultrasonography revealed parenchymal ischemic changes following BSI, and PV thrombosis developed. The BSI did not resolve to antibiotic treatment. Patient 6 encountered BSI 26 days after PLT. Similar to Patient 2, there was no abnormal perfusion in Doppler ultrasonography before or immediately after the BSI, but PV stenosis emerged afterward. Patient 11 experienced a BSI resistant to antibiotics, occurring 5 days after PLT. Following BSI, Doppler ultrasonography readings indicated an increase in the hepatic arterial (HA) Doppler ultrasonography resistive index (RI) value, confirming parenchymal ischemic changes, and HA thrombosis occurred. This HA thrombosis was resolved with medication alone, without operation or intervention. In the case of Patient 12, BSI with resistance to antibiotics was confirmed on the same day after PLT. There were no abnormal Doppler ultrasonography perfusion findings immediately post-BSI, but HA stenosis occurred. Finally, Patient 13 developed BSI 6 days after PLT, confirming an HV monophasic waveform on Doppler ultrasonography and subsequent HV stenosis.

## 4. Discussion

Postoperative BSI is a significant concern following major surgeries, including liver transplantation. This can lead to severe, life-threatening complications after PLT. The incidence and mortality of bacteremia following PLT have been the subjects of numerous studies [3,18,19,20,21,22]. Several factors influence the risk of BSI, including the patient’s immune status, surgical technique, perioperative care, and underlying predisposing conditions. Common pathogens associated with postoperative BSI in transplant recipients include *Staphylococcus aureus*, *Enterococcus* spp., and Gram-negative organisms [19,20].

This study aimed to validate the hypothesis that postoperative BSI can trigger early vascular complications and impair outcomes in patients with BA undergoing PLT. This study is the first to investigate whether BSI has an impact on postoperative vascular complications in patients with BA who underwent PLT. BSI can directly affect endothelial cells and the inner lining of blood vessels. Endothelial cells can be activated or damaged when exposed to bacterial toxins or inflammatory mediators during infection. Recent research supports this hypothesis by highlighting the role of bacterial toxins in intravascular coagulation and vascular dysfunction [13,14,15,16,17,23].

Coagulation and vascular complications related to infectious diseases have been reported in cases of sepsis due to Gram-negative and Gram-positive bacteria and non-bacterial pathogens such as viruses, protozoa (e.g., malaria), fungi, and spirochetes [23]. During sepsis, interactions between pathogen-associated patterns and pattern recognition receptors, including Toll-like receptors on immune cells, can trigger the release of various cytokines, leading to microvascular thrombosis [17]. Animal models have shown that staphylococcal cytotoxins can upregulate metalloprotease activity on endothelial cells, causing the cleavage of endothelial–cadherin and a loss of endothelial barrier function [14]. Other studies have indicated that bacteria and bacterial toxins establish intimate interactions with endothelial cells, triggering inflammatory responses and coagulation processes, modifying endothelial cell plasma membranes and junctions to attach to surfaces, and invading, crossing, and even destroying the endothelial barrier [15,16]. In infectious diseases, certain organs, such as the kidneys, are affected by intravascular coagulation and vascular dysfunction due to pathogen-induced inflammation [13,24,25]. Other animal studies have supported an association between vascular endothelial inflammation and vascular dysfunction or complications [26,27].

Early vascular complications following PLT are often attributed to surgical factors such as anastomotic stenosis, small vessel diameter, graft size mismatch, and anastomotic complexity [3,11,28,29,30]. The most common vascular event after PLT is hepatic artery thrombosis, which occurs in approximately 8% of cases, whereas portal vein thrombosis affects 5–10% of cases. Risk factors for vascular complications after post-PLT include recipient age, anastomotic anatomy, rejection, recipient hypotension, and hypercoagulability. The hypoplastic portal vein in BA recipients is a significant risk factor in portal vein thrombosis [3,11]. This study performed univariate and multivariate analyses with short-term vascular complications as the outcome variable, identifying GRWR and bacteremia within 1 month as risk factors for post-PLT vascular complications (Table 5). Among other factors, patient weight and complex vascular reconstruction were not significantly associated with vascular complications. This finding suggests that postoperative BSI is crucial for developing early vascular complications after PLT.

In this study, liver graft vascular flow was routinely assessed using Doppler ultrasonography after PLT. Among the 13 patients who developed BSI, vascular complications occurred in six. Importantly, except for two patients (P3 and P11), no abnormal perfusion findings were observed on Doppler ultrasonography before the onset of BSI in these patients (Table 6). These results show that vascular complications may occur after BSI in patients with normal graft perfusion without vascular stenosis or thrombosis due to surgical factors. While these cannot definitively establish BSI as the sole cause of vascular complications over surgical factors, they strongly suggest its significant contribution.

A previous study at the same institution reported a high incidence of cholangitis after KPE in BA patients. This suggests the need for empirical antibacterial therapy as enterococci are common pathogens in cholangitis after KPE [4]. Other studies have shown that home intravenous antibiotic therapy (HIVA) can effectively treat intractable cholangitis after KPE for BA [31,32]. Patients with intractable recurrent cholangitis, an indication for PLT, often experience BSI despite long-term empirical antibacterial therapy. In a study in which HIVA was performed for intractable cholangitis after KPE for BA, BSI was confirmed in the treatment group, and some patients who underwent PLT died from sepsis [32]. Although there was no significant outcome for the 3-year overall survival in this study, it revealed a significantly higher frequency of early vascular complications and reoperations in BA patients with BSI.

Our study has some limitations, primarily due to its retrospective nature, which limits our ability to confirm the detailed impact of surgical techniques and BSI strains on surgical outcomes. Although there have been studies on a few strains, such as *Staphylococcus*, basic biochemical investigations on the relationship between BSI and vascular damage are still in progress. Nonetheless, this study provides valuable clinical insights into the relationship between BSI and vascular complications in patients with BA undergoing PLT.

In conclusion, although challenging, proper management of bacterial infections and timely liver transplantation prior to the development of uncontrolled cholangitis may prove beneficial in reducing vascular complications and unexpected reoperation in patients with BA.

## Figures and Tables

**Table 1:** Baseline preoperative characteristics of liver transplant recipients with BA with or without postoperative bacteremia within 1 month.

	All PLTs (*n* = 67)	BT (*n* = 13)	NBT (*n* = 54)	*p*-Value
Age, years	1.08 (0.67–5.33)	0.75 (0.5–1.79)	1.17 (0.67–7.00)	0.098
Female	40 (59.7%)	8 (61.5%)	32 (59.3%)	0.880
Weight, kg	10.1 (8.0–17.5)	8.6 (7.0–13.2)	10.8 (8.0–22.8)	0.086
Presence of recurrent cholangitis before PLT (>2 admissions/year)	24 (35.8%)	7 (53.8%)	17 (31.5%)	0.197
History of bacteremia before PLT within 1 month	6 (9.0%)	3 (23.1%)	3 (5.6%)	0.082
Presence of bacteremia at the time of PLT	2 (3.0%)	2 (15.4%)	0 (0%)	0.035 *
WBC (μL)	6330 (4650–10,530)	10,530 (6435–15,025)	5955 (4543–8933)	0.006 *
Hemoglobin (g/dL)	10.2 (8.9–11.2)	10.1 (9.2–10.8)	10.2 (8.8–11.3)	1.000
Platelet (103/μL)	132 (95–181)	167 (80–215)	130 (95–181)	0.570
AST (IU/L)	147 (66–224)	185 (89–387)	145 (66–211)	0.186
ALT (IU/L)	46 (27–122)	46 (22–140)	45 (27–123)	0.842
Total bilirubin (mg/dL)	7.2 (3.1–13.1)	10.9 (6.1–18.8)	6.1 (2.7–11.8)	0.046 *
Protein (g/dL)	5.9 (5.0–6.5)	6.0 (4.8–6.5)	5.9 (5.1–6.5)	0.585
Albumin (g/dL)	3.3 (3.0–3.7)	3.3 (3.0–3.9)	3.3 (3.0–3.7)	0.553
Creatinine (mg/dL)	0.20 (0.20–0.30)	0.20 (0.20–0.47)	0.20 (0.20–0.29)	0.568
C-reactive protein (mg/L)	9.05 (2.62–19.53)	12.0 (3.2–76.3)	8.1 (2.1–17.3)	0.174
INR	1.27 (1.12–1.52)	1.50 (1.21–2.00)	1.26 (1.11–1.37)	0.039 *
MELD/PELD score	10.4 (1.3–16.0)	15.4 (9.9–23.2)	9.0 (1.2–15.4)	0.037 *
Admission status at the time of PLT				0.052
Outpatient	27 (40.3%)	3 (23.1%)	24 (44.4%)	
Inpatient	32 (47.8%)	6 (46.2%)	26 (48.1%)	
Intensive care unit	8 (11.9%)	4 (30.8%)	4 (7.4%)	
Follow-up period, months	45 (21–76)	46 (15–96)	45 (21–77)	0.885

Data are presented as median (interquartile range) or number (%). ALT, alanine aminotransferase; AST, aspartate aminotransferase; BT, recipients who experienced post-PLT BSI; INR, international normalized ratio; MELD, model for end stage liver disease; NBT, recipients without post-PLT BSI; PELD, pediatric end stage liver disease model; PLT, pediatric liver transplantation; WBC, white blood cell. * Statistically significant results.

**Table 2:** Donor and operative characteristics.

	All PLTs (*n* = 67)	BT (*n* = 13)	NBT (*n* = 54)	*p*-Value
Donor age	31 (25–37)	26 (23–33)	32 (27–38)	0.039 *
Donor graft weight (g)	283 (222–345)	283 (207–337)	283 (224–351)	0.587
GRWR	2.40 (1.69–3.12)	3.04 (2.38–3.77)	2.22 (1.48–3.01)	0.047 *
Graft type				0.508
Lateral segment	52 (77.6%)	12 (92.3%)	40 (74.1%)	
Left lobe	8 (11.9%)	0	8 (14.8%)	
Right lobe	3 (4.5%)	0	3 (5.6%)	
Whole liver	3 (4.5%)	1 (7.7%)	2 (3.7%)	
Reduced or monosegment	1 (1.5%)	0	1 (1.9%)	
Deceased donor	21 (31.3%)	5 (38.5%)	16 (29.6%)	0.74
Complex vascular reconstruction	11 (16.4%)	1 (7.7%)	10 (18.5%)	0.677

Data are presented as median (interquartile range) or as number (%). Numbers in bold indicate statistically significant results. BT, recipients who experienced post-PLT BSI; GRWR, graft-to-recipient weight ratio; NBT, recipients without post-PLT BSI; PLT, pediatric liver transplantation. * Statistically significant results.

**Table 3:** Clinical complications and outcomes after PLT.

	All PLTs (*n* = 67)	BT (*n* = 13)	NBT (*n* = 54)	*p*-Value
Portal vein stenosis	4 (6.0%)	2 (15.4%)	2 (3.7%)	0.167
Portal vein thrombosis	6 (9.0%)	2 (15.4%)	4 (7.4%)	0.329
Hepatic artery stenosis	4 (6.0%)	1 (7.7%)	3 (5.6%)	1
Hepatic artery thrombosis	2 (3.0%)	1 (7.7%)	1 (1.9%)	0.353
Hepatic vein stenosis	3 (4.5%)	1 (7.7%)	2 (3.7%)	0.482
Any type of vascular complications	16 (23.9%)	6 (46.2%)	10 (18.5%)	0.046 *
Biliary complications	9 (13.4%)	2 (15.4%)	7 (13.0%)	1
All types of complication	20 (29.9%)	6 (46.2%)	14 (25.9%)	0.185
Ventilator dependence, days	2 (1–5)	3 (1–9)	2 (1–4)	0.607
ICU stay, days	4 (3–8)	5 (4–18)	4 (3–6)	0.063
Hospital stay, days	25 (20–33)	31 (25–61)	24 (19–32)	0.107
Re-laparotomy	6 (9.0%)	4 (30.8%)	2 (3.7%)	0.011 *
Total bilirubin within 1 year (mg/dL)	0.50 (0.30–0.68)	0.40 (0.20–0.60)	0.50 (0.30–0.70)	0.316
Albumin within 1 year (g/dL)	4.1 (3.8–4.3)	3.9 (2.9–4.1)	4.1 (3.9–4.3)	0.036 *
AST within 1 year (IU/L)	32 (26–38)	35 (23–48)	38 (25)	0.622
ALT within 1 year (IU/L)	20 (14–27)	23 (11–41)	23 (14)	0.489
INR within 1 year	1.14 (1.07–1.23)	1.15 (1.08–1.25)	1.14 (1.06–1.22)	0.532
3-year graft survival	79.80%	76.9%	80.5%	0.495
3-year overall survival	81.30%	84.6%	80.5%	0.789

Data are presented as median (interquartile range) or as number (%). ALT, alanine aminotransferase; AST, aspartate aminotransferase; BT, recipients who experienced post-PLT BSI; ICU, intensive care unit; INR, international normalized ratio; NBT, recipients without post-PLT BSI; PLT, pediatric liver transplantation. * Statistically significant results.

**Table 4:** Microorganisms identified in bacterial infections.

Pathogens	Number of Episodes (%)	Source of Infection
Gram-positive		
Staphylococcus aureus	3 (17.6)	Intra-abdominal
Staphylococcus epidermidis	3 (17.6)	Catheter
Enterococcus faecium	4 (23.5)	Intra-abdominal
Gram-negative		
Acinetobacter bereziniae	1 (5.9)	Catheter
Herbaspirillum aquaticum	1 (5.9)	Catheter
Enterobacter cloacae	1 (5.9)	Biliary tract
Klebsiella pneumoniae	1 (5.9)	Intra-abdominal
Escherichia coli	1 (5.9)	Biliary tract
Burkholderia cepacia	1 (5.9)	Gastrointestinal tract
Pseudomonas aeruginosa	1 (5.9)	Respiratory tract

Biliary tract: pathogens collected from a drainage tube connected to the intra-abdominal space with suspected biliary leakage. Catheter: pathogens collected from intravenous catheters. Gastrointestinal tract: pathogens identified in the blood after a sudden onset of gastrointestinal symptoms. Intra-abdominal: pathogens collected from a drainage tube connected to the intra-abdominal space. Respiratory tract: pathogens collected from sputum during a suspected respiratory tract infection.

**Table 5:** Univariate and multivariate analyses of short-term vascular complications as outcome variables.

Variable	Univariate Analysis		Multivariate Analysis	
	OR (95% CI)	*p*	OR (95% CI)	*p*
Weight < 10 kg	2.381 (0.750–7.562)	0.141	0.560 (0.114–2.751)	0.475
History of bacteremia before LT within 1 month	0.613 (0.066–5.674)	0.667		
Bacteremia after LT within 1 month	3.771 (1.040–13.682)	0.043	5.691 (1.071–30.228)	0.041 *
GRWR > 4	15.600 (1.566–155.410)	0.019	27.214 (1.984–373.252)	0.013 *
DDLT vs. LDLT (reference: LDLT)	0.994 (0.296–3.339)	0.993		
Complex vascular reconstruction	2.095 (0.525–8.365)	0.295		
MELD/PELD > 20	1.731 (0.380–7.891)	0.479		
ABO-incompatible (reference: ABO-compatible)	1.314 (0.229–7.530)	0.759		

Numbers in bold indicate statistically significant results. CI, confidence interval; DDLT, deceased donor liver transplantation; GRWR, graft-to-recipient weight ratio; LDLT, living donor liver transplantation; LT, Liver transplantation; MELD, model for end stage liver disease; OR, odds ratio; PELD, pediatric end stage liver disease model. * Statistically significant results.

**Table 6:** Vascular characteristics of Doppler ultrasonography and antibiotic treatment in BT patients (*n* = 13).

Patient Number	Post-PLT Days When BSI Occurred	Doppler Days before BSI	Doppler Days after BSI	HA RI Value before BSI	HA RI Value after BSI	HV Phase before BSI	HV Phase after BSI	Perfusion Status	Pathogens of BSI	Resistance for Antibiotics	Resolved by Antibiotics	Antibiotics	Vascular Complications	Treatment by Intervention or Operation
P1	4	−1	3	0.71	NA	Triphasic	Triphasic	No abnormal perfusion before and after BSI	Staphylococcus epidermidis	No	Yes	Teicoplanin	No	No
P2	5	−2	2	0.84	0.62	Triphasic	Triphasic	No abnormal perfusion before and after BSI	Enterococcus faecium	No	Yes	Teicoplanin	PV stenosis PV thrombosis	Yes
P3	2	−1	1	0.63	NA	Triphasic	Triphasic	Parenchymal ischemic change after BSI	Staphylococcus aureus	No	No	Vancomycin	PV thrombosis	Yes
P4	6	−2	3	0.73	0.7	Triphasic	Triphasic	No abnormal perfusion before and after BSI	Staphylococcus aureus, Staphylococcus epidermidis	No	Yes	Teicoplanin	No	No
P5	5	−1	2	0.59	0.77	Triphasic	Triphasic	No abnormal perfusion before and after BSI	Enterococcus faecium	No	Yes	Teicoplanin	No	No
P6	26	−1	6	NA	NA	Triphasic	Triphasic	No abnormal perfusion before and after BSI	Staphylococcus epidermidis	No	Yes	Teicoplanin	PV stenosis	Yes
P7	6	−2	1	0.7	0.56	Triphasic	Triphasic	No abnormal perfusion before and after BSI	Enterococcus faecium	No	Yes	Teicoplanin	No	No
P8	5	−2	1	0.67	0.69	Triphasic	Triphasic	No abnormal perfusion before and after BSI	Staphylococcus aureus	No	No	Teicoplanin	No	No
P9	15	−3	4	0.75	0.78	Triphasic	Triphasic	No abnormal perfusion before and after BSI	Acinetobacter bereziniae, Herb spirillum aquaticum	Yes	Yes	Colistin	No	No
P10	0	No	1	NA	0.78	NA	Biphasic	No abnormal perfusion after BSI	Enterobacter cloacae	Yes	Yes	Amikacin, Teicoplanin	No	No
P11	5	−3	4	0.6	0.8	Triphasic	Triphasic	Parenchymal ischemic change after BSI	Klebsiella pneumoniae (ESBL positive)	Yes	Yes	Amikacin	HA thrombosis	No
P12	0	No	1	NA	0.75	NA	Triphasic	No abnormal perfusion after BSI	Escherichia coli, Burkholderia cepacia	Yes	Yes	Tigecycline	HA stenosis	Yes
P13	6	−2	1	0.71	0.72	Triphasic	Monophasic	No abnormal perfusion before and after BSI	Pseudomonas aeruginosa	No	Yes	Amikacin	HV stenosis	Yes

BSI, bloodstream infection; BT, recipients who experienced post-PLT BSI; Doppler days, number of days when Doppler ultrasonography was performed based on the time of BSI occurrence; ESBL, extended-spectrum beta-lactamases; HA RI, hepatic arterial Doppler ultrasonography resistive index; HV phase, hepatic venous Doppler ultrasonography waveform phase; NA, not available; PLT, pediatric liver transplantation; PV, portal vein.

## Data Availability

Not applicable.
